# CDC field triage criteria accurately predicts outcomes in high impact trauma

**DOI:** 10.5249/jivr.v14i1.1650

**Published:** 2022-01

**Authors:** Mason Charles Sifford, R. Dailey, R. Reif, M. Hutchison, C. Mason, K. Kimbrough, B. Davis, A. Bhavaraju, H. K Jensen, R. Robertson, J. Taylor, W.C. Beck, Kevin Sexton

**Affiliations:** ^*a*^ Trauma and Acute Care Surgery, University of Arkansas for Medical Sciences, Little Rock, AR, USA.; ^*b*^ Metropolitan Emergency Medical Services, Little Rock, AR, USA.

**Keywords:** Emergency Medical Services, Injury Severity Score, Triage, Retrospective study

## Abstract

**Background:**

The precision of emergency medical services (EMS) triage criteria dictates whether an injured patient receives appropriate care. The trauma triage protocol is a decision scheme that groups patients into triage categories of major, moderate and minor. We hypothesized that there is a difference between trauma triage category and injury severity score (ISS).

**Methods:**

This retrospective, observational study was conducted to investigate a difference between trauma triage category and ISS. Bivariate analysis was used to test for differences between the subgroup means. The differences between the group means on each measure were analyzed for direction and statistical significance using ANOVA for continuous variables and chi square tests for categorical variables. Logistic and linear regressions were performed to evaluate factors predicting mortality, ICU length of stay.

**Results:**

With respect to trauma triage category, our findings indicate that minor and moderate triage categories are similar with respect to ISS, GCS, ICU LOS, hospital LOS, and mortality. However, after excluding for low impact injuries (falls), differences between the minor and moderate categories were evident when comparing to ISS, GCS, ICU LOS, and hospital LOS. Additionally, after excluding for low impact injures, ISS, ICU LOS, and hospital stay were found to correlate well with trauma triage category.

**Conclusions:**

In this retrospective, observational study significant differences were not seen when comparing ISS with the trauma triage categories of moderate and minor during our initial analysis. However, a difference was found after excluding for low impact injuries. These findings suggest that CDC criteria accurately predicts outcomes in high impact trauma.

## Introduction

Emergency medical services (EMS) play a vital role in the determination of appropriate care for injured patients. EMS triage criteria assess the physiology, anatomy, and mechanism of an injury. Moreover, the precision of triage criteria dictates whether an injured patient receives the appropriate level of care for a given injury.^1^ Effective triage is vital to timely arrival at facilities capable of definitive care and maximizing survival. Specifically, evidence indicates that transfer of severely injured patients to hospitals that cannot provide definitive care is associated with an increase in mortality.^2^

**Figure 1:**
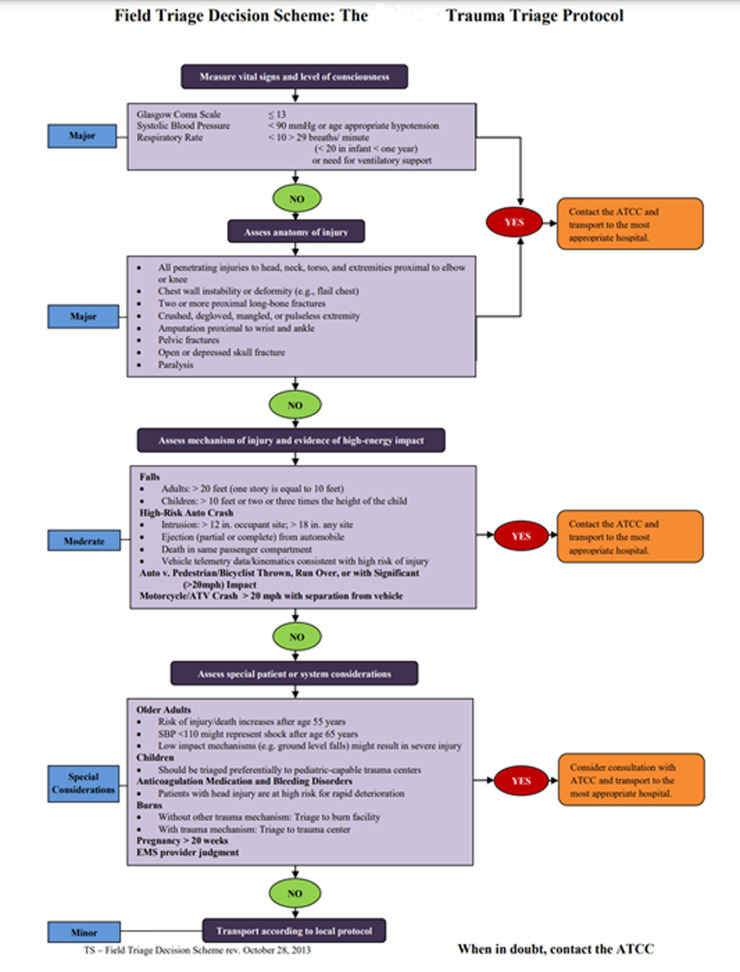
State Trauma Triage Protocol.

Injury Severity Score (ISS) is an established method for predicting trauma mortality, morbidity, and length of hospital stay after trauma.^1^ Arkansas’s Trauma Triage Protocol is a field triage decision tool based on CDC triage criteria that groups trauma patients into major, moderate, and minor categories. When EMS responds to a trauma, they evaluate vital signs, level of consciousness, and the anatomy of injury to determine the patient’s category. If criteria for major trauma are not met, the mechanism of injury and evidence of high-energy impact is assessed to determine if the patient should be placed in the moderate trauma category. If none of these criteria are met, the patient is deemed a minor trauma patient. Special considerations are made for burn victims and children. These categories are then used by EMS to make appropriate transportation decisions in the field. A full description of criteria and triage descriptions can be found in Figure 1.

Our study sought to examine any differences between trauma triage category and ISS and, if so, whether these differences could help restructure EMS field triage criteria to improve triage of patients, thereby improving patient outcomes. 

## Methods 

**Study setting**

After we obtained IRB approval, all trauma patients within the central region of Arkansas who were triaged and treated at a level 1 trauma center were identified in the local trauma registry. For the year of 2016, a total of 320 patients met the inclusion criteria. 

**Study design**

We conducted a retrospective, observational study to investigate the predictive differences between trauma triage category and ISS. The initial data set consisted of 536 patients, all of which were transported to a level 1 trauma center. Patients missing an assigned trauma triage category (n=4), ISS (n=11), NISS (n=11), or TRISS (n=23) were excluded from our analysis. After our initial analysis, we then excluded patients who had a mechanism of injury due to a fall (n=208). This group of patients heavily populated the minor trauma category (90% were in the minor trauma category) and geriatric age group (83%). This subgroup was excluded because although falls result in a low impact mechanism of injury, there is a significant chance for injury given the typical population usually affected. Numerous studies have identified the difficulties of triaging geriatric patients.^3,4,5,6^ Additionally, patients with a chief complaint of burn (n=16) were excluded leaving the final sample size of 320.

**Statistical analysis**

Descriptive statistics were used to summarize the data. Bivariate analysis was used to test for differences between the subgroup means for patients assigned to major, moderate, or minor trauma triage categories. Table 1 identifies the categorizations of Scores for Analysis. The differences between the group means on each measure were analyzed for direction and statistical significance using ANOVA for continuous variables and chi-square tests for categorical variables. Logistic regressions were performed to evaluate factors predicting mortality and ICU stay. Statistical significance was set at α=0.05 for all analyses. The analysis was conducted using SAS 9.4 and Stata 15. 

**Table 1:** Categorizations of Scores for Analysis.

Severe ISS	> 15
Glasgow Coma Scale	Severe (<9), Moderate (9-12), Mild (>12)
Geriatric patient	Age > 64
Low pre-hospital (on scene) systolic blood pressure (SBP)	<90 mmHg

## Results

Table 2 summarizes the descriptive statistics of the entire population before and after exclusions. The “before” data set consists of 528 patients with an average age of 50.4 years, 65.0% males, 52.4% white, and 94.1% survived. Prior to arriving to the hospital, the sample had a mean systolic blood pressure (SBP) of 131.9 mmHg, pulse rate of 90.0 bpm, and respiratory rate of 18.2 breaths per minute, GCS of 13.8 and 58.3% were assigned to the minor trauma triage category. The mean ISS of the sample was 9.3 with only 19.3% classified as having a severe injury (ISS > 15). The sample had a length of stay (LOS) of 3.9 days, stayed in the intensive care unit for approximately 1.1 days, and were on the ventilator for approximately 0.67 days. A majority of the population were either discharged home (76.8%) or transferred to a skilled nursing facility (10.0%). 

**Table 2:** Entire Population before and after exclusion.

Before		After	
Population	Descriptive Statistics	Population	Descriptive Statistics
Age, y	50.4 ± 21.7	Age, y	39.7 ± 15.8
Geriatric, n (%)	141 (27.8%)	Geriatric, n (%)	24 (7.5%)
Male, n (%)	330 (65.0%)	Male, n (%)	240 (75.0%)
White, n (%)	266 (52.4%)	White, n (%)	123 (38.4%)
Black or African American, n (%)	217 (42.7%)	Black or African American, n (%)	175 (54.7%)
**Mechanism of Injury**		**Mechanism of Injury**	
Blunt	415 (81.7%)	Blunt	227 (70.9%)
Penetrating	93 (18.3%)	Penetrating	93 (29.1%)
**Trauma Triage Category**		**Trauma Triage Category**	
Major, n (%)	122 (24.0%)	Major, n (%)	115 (35.9%)
Moderate, n (%)	90 (17.7%)	Moderate, n (%)	77 (24.1%)
Minor, n (%)	296 (58.3%)	Minor, n (%)	128 (40.0%)
Pre-hospital GCS	13.8 ± 2.9	Pre-hospital GCS	13.6 ± 3.3
ISS	9.3 ± 10.2	ISS	10.4 ± 11.0
ISS > 15, n (%)	93 (19.3%)	ISS > 15, n (%)	72 (23.0%)
NISS	12.2 ± 13.8	NISS	13.9 ± 15.1
TRISS	0.94 ± 0.18	TRISS	0.92 ± 0.21
Pre-hospital SBP	131.9 ± 37.3	Pre-hospital SBP	125.4 ± 37.9
Pre-hospital Pulse	90.- ± 25.2	Pre-hospital Pulse	91.8 ± 26.6
Pre-hospital Respiratory Rate	18.2 ± 4.6	Pre-hospital Respiratory Rate	18.0 ± 5.3
Total Hospital Days	3.9 ± 6.0	Total Hospital Days	3.8 ± 6.3
Total ICU Days	1.1 ± 3.7	Total ICU Days	1.3 ± 3.9
Total Ventilation Days	0.67 ± 3.1	Total Ventilation Days	0.83 ± 3.3
Alive, n (%)	478 (94.1%)	Alive, n (%)	293 (91.6%)
**Discharge **		**Discharge**	
Home, n (%)	390 (76.8%)	Home, n (%)	266 (83.1%)
Dead, n (%)	30 (5.9%)	Dead, n (%)	27 (8.4%)
Rehab, n (%)	32 (6.3%)	Rehab, n (%)	14 (4.4%)
SNF, n (%)	51 (310.0%)	SNF, n (%)	11 (3.4%)
Hospital transfer, n (%)	4 (0.79%)	Hospital transfer, n (%)	2 (0.6%)
Hospital, n (%)	1 (0.20%)	Hospital, n (%)	0 (0%)

GCS: Glasgow Coma Scale; ISS: Injury Severity Score; NISS: New Injury Severity Score; TRISS: Trauma and Injury Severity Score; SBP: systolic blood pressure; Geriatric > 64; Mild GCS > 12; Severe GCS <9; Moderate GCS = 9-12; SNF: Skilled Nursing Facility)

The “after” columns summarizes the descriptive statistics of the population after excluding falls from the analysis. The data set consists of 320 patients with an average age of 39.7 years, 75.0% males, 38.4% white and 91.6% survived. Prior to arriving to the hospital, the sample had a mean systolic blood pressure (SBP) of 125.4 mmHg, pulse rate of 91.8 bpm, and respiratory rate of 18.0 breaths per minute, GCS of 13.6 and 35.9% of patients were assigned to the minor trauma triage category as the minor category was heavily populated by low impact falls. The mean ISS of this subset of the population was 10.4, with only 23.3% classified as having a severe injury (ISS > 15). On average, the sample had a length of stay of 3.8 days, stayed in the intensive care unit for 1.3 days, and were on the ventilator for approximately 0.83 days. A majority of the population were discharged home (83.1%) or died in-hospital (8.4%)

Table 3a represents the entire population of patients stratified by trauma triage category. Major, moderate and minor patients varied significantly in race (p=0.019), mortality (p<.0001), discharge status (p<.0001), and type of injury (p<.0001). Once stratified by trauma triage category, the patients varied significantly in age (age: 39.1 vs. 39.5 vs. 58.3, p<.0001; geriatric: 5.7% vs. 7.8% vs. 42.9%, p<.0001), ISS (ISS: 16.0 vs. 9.5 vs. 6.2, p <.0001; ISS >15: 41.0% vs. 22.5% vs. 8.5%), and pre-hospital GCS (GCS: 11.6 vs. 14.1 vs. 14.7, p<.0001; GCS categories: 64.8% vs. 92.2% vs. 98.0%, p<.0001). Additionally, once stratified by trauma triage category, patients also varied significantly in NISS (p<.0001), TRISS (p<.0001), and pre-hospital SBP (p<.0001), pulse rate (p=.0022), and respiratory rate (p=.005). 

**Table 3a:** Entire Population; Trauma Triage Category: Major, Moderate, Minor (n = 508)

Population	Major (n = 122)	Moderate (n = 90)	Minor m (n = 296)	P
Age, y	39.1 ± 14.7	39.5 ± 16.8	58.3 ± 22.0	**<.0001**
Geriatric	7 (5.7%)	7 (7.8%)	127 (42.9%)	**<.0001**
Male	106 (87.0%)	64 (71.1%)	160 (54.1%)	**<.0001**
White	42 (34.4%)	44 (49.0%)	180 (60.8%)	
Black or AA	72 (59.0%)	44 (49.0%)	101 (34.1%)	**0.0187**
Other	8 (6.6%)	2 (2.2%)	15 (5.1%)	
Dead	28 (23.0%)	0 (0%)	2 (0.7%)	
Home	78 (64.0%)	78 (86.7%)	234 (79.1%)	
Hospital transfer	2 (1.6%)	1 (1.1%)	1 (0.3%)	**<.0001**
Hospital	0 (0%)	0 (0%)	1 (1.1%)	
Rehab (Inpatient)	8 (6.6%)	6 (6.7%)	18 (6.1%)	
SNF	6 (4.9%)	5 (5.6%)	40 (13.5%)	
Dead	28 (23.0%)	0 (0%)	2 (0.68%)	**<.0001**
Alive	94 (77.1%)	90 (100.0%)	294 (99.3%)	
Blunt	53 (43.4%)	81 (90.0%)	281 (94.9%)	**<.0001**
Penetrating	69 (56.6%)	9 (10.0%)	15 (5.1%)	
Total hospital days	5.8 ± 9.1	4.2 ± 6.0	3.0 ± 4.0	**<.0001**
Total ICU days	3.0 ± 6.5	0.98 ± 2.7	0.44 ± 1.6	**<.0001**
Total Vent days	2.1 ± 5.1	0.56 ± 3.9	0.10 ± 0.52	**<.0001**
Pre hospital pulse	85.1 ± 37.2	94.7 ± 18.1	90.5 ± 20.3	**0.0224**
Pre hospital respiratory rate	17.0 ± 7.8	18.5 ± 3.3	18.6 ± 2.7	**0.0047**
Pre hospital GCS	11.6 ± 4.8	14.1 ± 2.3	14.7 ± 0.75	**<.0001**
Mild GCS	79 (64.8%)	83 (92.2%)	290 (98.0%)	
Moderate GCS	10 (8.2%)	3 (3.3%)	4 (4.4%)	**<.0001**
Severe GCS	30 (27.1%)	4 (4.4%)	1 (0.3%)	
ISS > 15	50 (41.0%)	20 (22.5%)	23 (8.5%)	**<.0001**
ISS	16.0 ± 15.0	9.5 ± 7.5	6.2 ± 6.1	**<.0001**
NISS	21.6 ± 20.2	11.9 ± 9.5	8.2 ± 8.3	**<.0001**
TRISS	0.81 ± 0.32	0.98 ± 0.042	0.98 ± 0.028	**<.0001**
Pre-hosp SBP	105.9 ± 48.9	132.6 ± 24.7	141.8 ± 29.8	**<.0001**

ED: Emergency department; Glasgow Coma Scale; ISS: Injury Severity Score; NISS: New Injury Severity Score; TRISS: Trauma and Injury Severity Score; SBP: systolic blood pressure; Geriatric > 64; Mild GCS > 12; Severe GCS <9; Moderate GCS = 9-12; SNF: Skilled Nursing Facility; TTA: Trauma Team Activation

Table 3b represents patients stratified by trauma triage category after excluding for falls. Major, moderate and minor patients who did not fall varied significantly in race (p=0.019), mortality (p<.0001), discharge status (p<.0001), and type of injury (p<.0001). Once stratified by trauma triage category, the patients varied significantly in ISS (ISS: 15.3 vs. 9.9 vs. 6.1, (p<.0001; ISS >15: 62.5% vs. 25.0% vs. 12.5%), (p<.0001) and pre-hospital GCS (GCS: 11.7 vs. 14.3 vs. 14.8, (p<.0001; GCS categories (mild): 27.7% vs. 26.3% vs. 46.0%, (p<.0001). Significant differences were also found in NISS (p<.0001), TRISS (p<.0001), and pre-hospital SBP (p<.0001), pulse rate (p=.009) and respiratory rate (p=.016).

**Table 3b:** Fall excluded Population; Trauma Triage Category: Major, Moderate, Minor (n = 320)

	Major (n = 115)	Moderate (n = 77)	Minor (n = 128)	P
Age, y	38.6 ± 14.5	37.5 ± 15.1	41.9 ± 17.0	**0.1063**
Geriatric	7 (29.2%)	4 (16.7%)	13 (54.2%)	**0.3291**
Male	99 (41.2%)	58 (24.2%)	83 (34.6%)	**0.001**
Race				
White	36 (29.3%)	35 (28.5%)	52 (42.3%)	
Black or AA	71 (40.6%)	41 (23.4%)	63 (36.0%)	**0.0416**
Other	8 (36.4%)	1 (4.6%)	13 (59.1%)	
Discharge Status				
Dead	27 (100.0%)	0 (0%)	0 (0%)	
Home	78 (29.3%)	68 (25.6%)	120 (45.1%)	
Hospital transfer	1 ( 50.0%)	0 (0%)	1 (50.0%)	**<.0001**
Rehab (Inpatient)	5 (35.7%)	6 (42.9%)	3 (21.4%)	
SNF	4 (36.4%)	3 (27.3%)	4 (36.4%)	
Alive	88 (30.0%)	77 (26.3%)	128 (43.7%)	**<.0001**
Type of Injury				
Blunt	46 (20.3%)	68 (30.0%)	113 (49.8%)	**<.0001**
Penetrating	69 (74.2%)	9 (9.7%)	15 (16.1%)	
Total hospital days	5.3 ± 8.7	4.1 ± 5.6	2.2 ± 2.7	**0.0004**
Total ICU days	2.6 ± 5.8	1.0 ± 2.9	0.26 ± 0.97	**<.0001**
Total Vent days	1.8 ± 4.1	0.62 ± 4.2	0.10 ± 0.56	**0.0003**
	Major (n = 112)	Moderate (n = 76)	Minor (n = 127)	
Pre hospital pulse	85.8 ± 36.8	93.8 ± 18.5	96.0 ± 17.6	**.0094**
	Major (n = 110)	Moderate (n = 70)	Minor (n = 122)	
Pre hospital respiratory rate	16.9 ± 8.0	18.7 ± 2.9	18.7 ± 2.5	**0.0162**
Mild GCS	76 (27.7%)	72 (26.3%)	126 (46.0%)	**<.0001**
Moderate GCS	9 (69.2%)	3 (23.1%)	1 (7.7%)	
Severe GCS	30 (90.9%)	2 (6.1%)	1 (3.0%)	
TTA Level 1	93 ( 74.4%)	17 (13.6%)	15 (12.0%)	
TTA Level 2	21 (13.0%)	58 (35.8%)	83 (51.2%)	**<.0001**
TTA Level 3	1 (5.6%)	2 (11.1%)	15 (83.3%)	
TTA Level 4	0 (0%)	0 (0%)	7 (100.0%)	
ISS > 15	45 (62.5%)	18 (25.0%)	9 (12.5%)	**<.0001**
ISS	15.3 ± 14.7	9.9 ± 7.5	6.1 ± 5.6	**<.0001**
NISS	21.0 ± 20.3	12.2 ± 9.3	8.2 ± 7.7	**<.0001**
TRISS	0.82 ± 0.32	0.99 ± 0.045	0.99 ± 0.022	**<.0001**
	Major (n = 107)	Moderate (n = 76)	Minor (n = 127)	
Pre-hosp SBP	105.6 ± 48.6	130.7 ± 23.9	139.0 ± 25.7	**<.0001**

ED: Emergency department; Glasgow Coma Scale; ISS: Injury Severity Score; NISS: New Injury Severity Score; TRISS: Trauma and Injury Severity Score; SBP: systolic blood pressure; Geriatric > 64; Mild GCS > 12; Severe GCS <9; Moderate GCS = 9-12; SNF: Skilled Nursing Facility; TTA: Trauma Team Activation

Table 4a represents the likelihood of having an ICU stay (falls excluded) after controlling for ISS, age, gender, trauma triage category, pre-hospital pulse, injury type, race, and pre-hospital GCS. After controlling for all of these variables, patients with an ISS>15 are more likely to have a stay in the ICU compared to patients with an ISS is less than 15 (OR 10.56, p<.0001). Compared to non-geriatric patients, geriatric patients are more likely to have a stay in the ICU (OR 3.99, p =0.010). Compared to patients classified as major TTC, minor patients are less likely to have a stay in the ICU (OR 0.26, p=0.010), whereas being classified as moderate is not a predictor of an ICU stay (p=0.110). Gender, injury type, race, and pre-hospital pulse and GCS are not significant predictors of an ICU stay. Additionally, there was no significant interactions between trauma triage category and ISS. 

**Table 4a:** Likelihood of ICU stay.

Population	Odds Ratio	P	Confidence Interval
ISS > 15	10.56	0.00	4.98 - 22.41
Geriatric	3.99	0.01	1.34 - 11.93
Male	1.12	0.77	0.51 - 2.46
TTC: Moderate	0.64	0.33	0.26 - 1.57
TTC: Minor	0.26	0.01	0.10 - 0.68
Pre hospital Pulse	1.01	0.11	1.00 - 1.02
Penetrating	0.85	0.69	0.39 - 1.87
African American	1.02	0.96	0.52 - 2.00
Other	0.65	0.57	0.15 - 2.85
Pre Hospital GCS	1.01	0.88	0.90 - 1.13
_cons	0.08	0.00	0.01 - 0.45

Table 4b represents the likelihood of survival (falls excluded) after controlling for ISS, age, gender, pre-hospital pulse, injury type, race, and pre-hospital GCS. After controlling for all these variables, patients who had an ISS > 15 (OR 0.056, p = 0.008) are less likely to survive compared to patients with an ISS less than 15. Compared to non-geriatric patients, geriatric patients are less likely to survive (OR 0.016, p=.004). Penetrating patients are less likely to survive than blunt patients (OR 0.158, p=.039). The higher a patient’s GCS the higher the patient’s chance of surviving (OR 1.62, pp<.0001). Trauma triage categories were excluded from this regression because both moderate and mild categories perfectly predicted mortality.

**Table 4b:** Likelihood of Survival.

Population	Odds Ratio	P	Confidence Interval
ISS > 15	0.056	0.0080	0.007 - 0.475
Geriatric	0.016	0.0040	0.001 - 0.267
Male	0.02	0.0290	0.001 - 0.676
Pre hospital Pulse	1.024	0.0960	0.996 - 1.052
Penetrating	0.158	0.0390	0.027 - 0.910
African American	1.149	0.8840	0.176 - 7.525
Other	0.338	0.6510	0.003 - 37.013
Pre Hospital GCS	1.62	<.0001	1.282 - 2.047

## Discussion

This retrospective, observational study compared ISS to field-based trauma triage category. Our findings indicate that patient outcomes in minor and moderate trauma triage categories are similar with respect to ISS, GCS, ICU LOS, hospital LOS, and mortality. However, after excluding falls from analysis, differences between the moderate and minor categories were evident when comparing ISS, GCS, ICU LOS, and hospital LOS, meaning that the trauma triage category was effective in predicting the severity of injury when falls, which were primarily geriatric low-impact trauma, were eliminated.

 Previous studies evaluating EMS triage criteria have shown criteria to be relatively insensitive at identifying seriously injured patients.^7,8^ These findings were particularly evident in the geriatric population as they were found to be significantly under-triaged to tertiary trauma centers and often required an inter-hospital transfer.^7^ This is particularly important because there is research that suggests a decreased risk of death is associated with direct transport to a level I trauma center.^9^ Consequently, improving the predictive ability of EMS field triage criteria has the potential to not only improve patient care and outcomes, but also decrease the need and cost of inter-hospital transfers. Studies have suggested that existing criteria could be revised to better identify seriously injured older adults at the expense of over-triage to major trauma centers.^8^ Our study found that after excluding falls, which were predominantly associated with the geriatric population, injury severity score, ICU LOS, and hospital stay correlated well with trauma triage category. Furthermore, our study showed that deaths only occurred in the major triage category, making it an excellent predictor of mortality, though this conclusion is severely limited by the low number of deaths in the database.

 Our findings support the fact that accurately triaging geriatric patients remains challenging.^3,4,5,6^ There are numerous possible reasons for the under-triage of elderly patients, including differences in physiologic response and increased comorbidities.^4^ The majority of falls excluded in our final analysis were low impact injuries among the elderly. These falls heavily populated the minor trauma category and skewed the differences between minor and moderate groups. After their exclusion, differences between minor and moderate categories were established and the criteria performed well. Thus, in centers where the field triage criteria have not been considered accurate enough, one should consider whether the low impact trauma in the elderly population is the reason for the underperformance in this regime. 

Our study has revealed that low impact trauma among the elderly may be a confounding factor that can help explain the contradictory reports about the sensitivity and specificity of the field triage criteria in accurately predicting the necessary response to trauma. A potential area for further study is to explore what methodology could improve the triage of elderly patients. One study demonstrated that the under-triage of elderly patients could be improved by the addition of elderly-specific guidelines to national triage guidelines.^8^ Another study demonstrated that Ohio’s geriatric-specific trauma triage guidelines provided superior sensitivity to that of the standard adult triage guidelines.^10^ Both studies had associated decreases in specificity and increases in over-triage; however, if improvement in the under-triage of elderly patients is desired, elderly specific guidelines should be considered. Because of this decreased specificity and increases in over-triage, it would be beneficial to develop new triage guidelines for low-impact injuries in the geriatric population instead of using the existing guidelines. In future studies, we plan to look specifically at the geriatric population in a more structured fashion and explore methodology that would improve the triage of elderly patients. It would be interesting to explore a prospective study where geriatric field triage criteria would be utilized in adults over 64 years of age for low-impact trauma. 

As with all retrospective studies, potential limitations include bias and confounders that cannot be accounted for in the statistical evaluation. Furthermore, this was a single-institution study and the results may not be generalizable to all institutions utilizing field-triage criteria, as there can be significant institutional or regional differences in patient populations, as well as differences in triage patterns. Aside from this, there were only 320 patients in the study population after excluding falls. This weakens its statistical power and the conclusions that can 

## Conclusion

In this retrospective, observational study, field trauma triage categories correlated well with differences in ISS, ICU and hospital length of stay after excluding low impact injuries, despite the lack of statistically significant differences when comparing ISS with the trauma triage categories of moderate and minor during our initial analysis. These findings suggest that CDC criteria accurately predict outcomes in high impact trauma and that low impact injuries, often seen in a geriatric population, may confound the analyses of the effectiveness of field triage protocols. This could identify the importance of creating specific geriatric guidelines for field triage criteria.

**Acknowledgments**

Our sincere gratitude to Ms. Judy Bennett for data support.
